# Prevalence of anxiety depression and stress among first year medical students in Tamilnadu

**DOI:** 10.6026/97320630019649

**Published:** 2023-05-31

**Authors:** Bama Rajanayagam, Sundaramahalingam Manikandan, Nag Anand, Sundaramoorthy Selvaraj

**Affiliations:** 1 Bharath Institute of Higher Education and Research, Selaiyur, Chennai, India; 2 Department of Physiology, Indira Medical College and Hospital, Thiruvallur, Tamilnadu, India; 3 Department of Physiology, Tagore Medical College and Hospital, Rathinamangalam, Chennai. India; 4 Department of General Medicine, Sree Balaji Medical College and Hospital, Chromepet, Chennai, India; 5 Department of Biochemistry, Indira Medical College and Hospital, Thiruvallur, Tamilnadu, India

**Keywords:** DASS (depression, anxiety and stress, salivary cortisol, medical students, Examination

## Abstract

The present study was to evaluate the examination stress of the first year MBBS students, prior to their university exam by assessing the mood parameters and cortisol level. A cross sectional study was conducted in 150 students of Indira Medical College, Thiruvallur from January to February 2022. The assessment methods implemented were Self-administered, pre-designed questionnaire of DASS 10 scale scoring 0-40, and salivary cortisol by using quantitative ELISA on relaxed (before exam) and stressed (on day of exam) students with prior consent. Respondent data were analysed using the independent t-test and Odds ratio logistic regression analysis was done for strength of association by using (SPSS) version 26.0 and the level of significance *p≤0.05. The prevalence of stress (43%), anxiety (35%), depression (22%) and level of cortisol (2.61±0.41 and 5.14±0.35) between relaxed and stressed respectively were significantly increased due to examination stress despite any significant change in academic performance. Odds ratio of stress (95% CI 2.153), anxiety (3.038), depression (2.513) and salivary cortisol (2.872) were significantly high in stressed. Female students were found to be more susceptible to stress than male students due to examination (p≤0.001**). This study suggests that the medical education and examination are unavoidable stressor in first year students. This could be prevented by providing stress reduction interventions and orientation programmes which could improve student mental well-being and reduce the psychological distress.

## Background:

Stress has become a ubiquitous part of our lives in recent times irrelevant of age. Measuring stress is difficult as there exists no definition of stress which is accepted by everyone. However, it is clear that stress in any form affects the bodily functions either physically or mentally and hinders the wellbeing of an individual [1]. Medical education is considered highly stressful by the students due to academic burden, poor planning and time management, peer pressure by teachers, high expectation from parents and lack of sleep [2,3]. When compared with the general public, a medical student's satisfaction in life and mental well-being is highly compromised [4]. And also competition in medical education, students are subjected to various stress factors such as failure apprehension, low scores in examinations and pressure to perform well in examinations. All these factors lead to stress in students and prevents them to perform well [5]. It causes palpitations, muscle weakness, fatigue, Dyspnoea, abdominal pain, headaches, and emotional repercussions such as apprehension, difficulty in concentration, feeling tense, temperament changes, impatience, nightmares, and disturbed sleep [6, 7]. Stress can also lead to cognitive symptoms such as confusion, impaired judgment, going blank, and difficulty in organizing thoughts [8]. This occasionally has an adverse effect on their mental health, as evidenced by 358 medical student suicides between 2010 and 2019; roughly 7 out of 10 victims were under the age of 30 in the five year's course duration [9].

Unfortunately, the COVID-19 pandemic had also adversely affected the functionality of educational institutions and mental health of student's routine activities which is not addressed appropriately. Lecture based teaching is simply transitioned to an online format, interactive small group sessions and clinical exposure are not well implicated [10]. Several studies have reported that medical training causes high incidences of psychological distress among students [11]. For instance, Alvi *et al.* [12] reported that 47.7% students suffered from anxiety, 35.1% depression, and 24.37% faced both, at some point of time during their MBBS tenure. A similarly high level of stress (49.9%) was reported by Oura *et al.* [13]. To address this defect on student's mental health the National Medical Commission (NMC), the governing body of the medical education system in India, has conceptualized the idea of a competency based medical education (CBME) system. This seeks to improve clinical relevance, academic load reduction, learning and evaluation in medical education [14, 15]. However, a high level of anxiety and depression was found among first year medical students due to lack of preparation and uncertainty over their examinations by pandemic situation and other factors. Academic stress has been considered as one of the most important factors as performance in examinations determines the future on student's career. Stress response is assessed by an increase in corticosteroid release. Some individuals show persistent or increased in cortisol level response to stress, while others show little or no such response depends on the levels of stress [16]. Generally Cortisol concentration and its rates of excretion increase in students during the period of examination [17]. Hence exams are their key concern, which causes more anticipation and stress before taking it up by the students. The Present study was undertaken to explore the effect of examination on their levels of stress, depression, anxiety and salivary cortisol in first year students proceeding to university examination.

## Material and Methods:

A cross-sectional study was conducted in the first year MBBS students at Indira medical college and Hospital, Thiruvallur Tamil Nadu, India, from January to February 2022. Institutional Human Ethical Committee approval (IECHS no: 13 October, 2021) was obtained and prior consent was taken from the participants included in the study. Inclusion criteria: 150 First year MBBS Medical students who were proceeding to university examination aged between 17 to 21 years and irrespective of gender, ages willing to participate were included in the study. Exclusion criteria: Students already diagnosed with any medical or psychiatry disorder were excluded from the study. Sample size: The sample size was derived based on study by Chaudari *et al.* [18] considering prevalence=32.8%, 5% absolute precision, 95% confidence interval and 5% allowable error by using the formula n= Zα2 PQ/d2 and all the 150 samples were collected for this study.

## Study Tool (Anxiety, Depression and Stress):

The participants were briefed about the study and recruited by a simple random sampling method. After the proper consent, questionnaire made up by Google forms was administered through social media platforms (Watsapp and Email) to the students a week before to the scheduled university final examination considered as (relaxed state), likewise on the day of examination (1st of February 2022) between 7.30 am to 8.30 am (Stressed state). The Self-administered, pre-tested anonymous questionnaire were used to collect the participant's socio-demographic and personal characteristics, DASS -10 item tool pre-designed questionnaire was used to measure the Stress, Depression and Anxiety developed by Sheldon Cohen [19,20]. These questionnaires were designed to assess the situation of how well the students were able to manage stress, anxiety and the burden of taking up exams in the last one month. For each question, students were made to choose their response from the questionnaire as, never/ almost never/ sometimes/ fairly often/ very often. Individual scores were assessed on the scale range from 0 to 40. Scores between 0 and 13 were considered to be a low, scores between 14 and 26 were considered moderate and the scores in the range of 27-40 indicate higher level of stress.

## Analysis of Salivary cortisol:

Salivary sample was assessed to evaluate the stress levels in students during the examination (Stressed state) and before the examination (Relaxed state). Students were instructed to collect the saliva sample with provided container in the morning (between 6.30 am to 7.30 am) a weak before the examination likewise on the day of university examination which had started from 1st of February 2022 on 9.30 am. Cortisol exhibits a large diurnal variation. Salivary cortisol concentrations increase in the morning, peaking at approximately 30 min after awakening in the morning and gradually decreasing throughout the day, so saliva sample were preferred to be collected in the morning. It was collected by using passive droll method as described by Granger *et al.*, [22] and stored at -20°C. After thawing, they were centrifuged for 10 minutes at 4000 rpm and the supernatants were collected and analysed with salivary cortisol enzyme immunoassay kit (SALIMETRICS) purchased from Thermo fisher. Assay buffer was used to dilute and all samples were assayed in triplicate. Cortisol was diluted using BSA stocks solution (1 mg/ ml) at pH 9.6. 200 microliter /well, was added to a 96 well plate and was incubated overnight at 4°C. The blocking buffer was added and samples were kept in the incubator for 2 hours at 37°C. After the washing, diluted primary antibody were added and kept incubated for 45 min at 37°C. Secondary antibody was added 100 µl/ well after the washing and was incubated for 30 minutes at 37°C. Streptavidin peroxidase solutions were added to the samples and incubated at 4°C for 15 minutes. Then the results were recorded with plate reader at 450 - 490 nm.

## Statistical Analysis:

The respondents data were collected and analysed using SPSS, version. 26.0. The descriptive data were analysed based on total scores (0-40) as those who had low, moderate and high Depression, Anxiety, stress and cortisol (ng/ml) level were expressed in mean and standard deviation (SD). Logistic Regression analysis was used to interpret odds ratios with a 95% confidence interval to assess the strength of associations between variables. The p-value ≤0.05* and ≤0.00** was considered significant for all the statistical analysis.

## Result

A total of 150 students were included in this study. The majority of students 83(55%) were female and 67(45%) were male. The mean age of the participants was 18.52±1.03 years. In our study population, hostellers were more in proportion 64% than day scholars 36% which showed in (Table 1).

[Table 2] shows DASS 10 scale score obtained from students on levels of Stress, anxiety, and depression during relaxed state were compared with stressed state. Out of 150 those who were 126 (84.12%) had mild depression, 18 (12.45%) had moderate depression, and 6 (4.39%) had severe or extremely severe depression. Among the study participants, 18 (12.42%) had mild anxiety, 96 (64.89%) had moderate anxiety, and 36(24.56%) had severe or extremely severe anxiety. Likewise 24 (16.28%) study participants had mild stress, 84 (56.72%) had moderate stress and 42 (28.22%) had severe or extremely severe stress. All the mood parameters were compared with male and female variables and which showed significant increases during examination in female participants than compare to male subjects p*≤0.05.

(Table 3): Logistic Regression analysis was done on moods parameters, blood pressure and cortisol levels among study participants on the day of exam (21.27±2.51, 24.06 ±4.01, 10.23±1.09, 5.14±0.35) (Stressed) and before exam (relaxed) (12.7±0.88, 11.27±1.40, 6.88±0.6, 2.61±0.40). Odds ratio 95% CI - (2.153, 3.038, 2.513, 2.872) shows significant association between the study participants p*≤0.05.

(Table 1Table 4) Mood parameters and salivary cortisol levels were compared between males and females. Female exhibited significantly higher baseline levels of anxiety (14.91±5.1, 19.72±4.8), stress (16.82±3.8, 21.27±5.1) and depression (7.61±3.4, 8.35±4.1) as compared to male. Both males and females showed an increase in the level of stress, anxiety and depression during exams compare to relaxed state (P≤0.00**). Likewise increase in cortisol levels (5.12±2.8 and 6.01±3.1) were observed in female students than male due to examination stress which is statistically significant.

## Discussion:

The capacity of adjustment to external stimuli is the most characteristic feature of life matter according to Seyle. Adaptation to our surrounding is one of the most important physiological reactions in life, if one doesn’t adapt it leads to stress [23]. Stress has a negative effect on cognitive function and thus affects learning capacity of the students. Medical studies are vast and difficult, that many studies have reported an increased level of stress in students with a prevalence ranging between 27-73%. Reports show stress and anxiety to be comparatively high in the first-year medical students [24]. This is because, Students post schooling encounter medical education system tough and rigid with a very vast syllabus that the environment itself becomes too taxing and highly competitive for them to thrive to perform and prove themselves. During which students develop palpitations, muscle weakness, fatigue, Dyspnoea, abdominal pain, headaches, and emotional repercussions such as apprehension, difficulty in concentration, feeling tense, temperament changes, impatience, nightmares, and disturbed sleep.

Hence this study was conducted to evaluate the level of stress among the first year medical students undergoing university examination and was correlated with their academic performance. The results showed significant changes in the levels of mood parameters 56% , stress 64% anxiety and increase in salivary cortisol (5.14±0.35) during the period of examination than in the students before examination (2.61 ±0.40). This shows that stress or anxiety had negligible impact on the student's performance in the exam with the percentile score of males and females being 75.5±12.6 and 77.27±10.9 respectively. This might be due to the adaptations that take place in the autonomic and central nervous system due to stress in due course of time.

A study conducted by Taneja *et al.*, [25] in Government medical college, New Delhi during September 2017 showed that 32% were depressed, 40.1% had anxiety, and 43.8% had stress. Similarly a study by Shah P [26] has reported that overall prevalence of depression, anxiety, and stress were found to be 44%, 59.3%, and 45.1% respectively in first-year medical students, Nepal Medical College and Teaching Hospital, Kathmandu, Nepal between June and July 2018. These findings are similar to the present study observations. A study in the neighbouring state of Kerala also showed higher depression scores among female students, which resembles the present study. The reason for the high prevalence of depression, anxiety, and stress among medical students can be attributed to various exposure factors such as high pressure to succeed, academic overwork, staying away from family members, and adjusting to clinical encounters.

Quince *et al.* performed a longitudinal study at a UK medical school and found a prevalence of depression ranging from 5.7% to 10.6% among students in the basic years and 2.7% to 8.2% in students from clinical stages of the course.[24] Quince *et al.* performed a longitudinal study at a UK medical school and found a prevalence of depression ranging from 5.7% to 10.6% among students in the basic years and 2.7% to 8.2% in students from clinical stages of the course.[24] Quince *et al.* performed a longitudinal study at a UK medical school and found a prevalence of depression ranging from 5.7% to 10.6% among students in the basic years and 2.7% to 8.2% in students from clinical stages of the course[24].

Further significant increase in salivary cortisol was observed in the students who have appeared for the examination (5.14±0.35) than the students before examination (2.61 ±0.40). This parallel increase in salivary cortisol level may be, in relevance with stress, anxiety faced by the students, due to lack of preparation, inadequate sleep or inability to cope up with the situation [29]. When a person faced with stress cannot cope up, the HPA axis is activated through the association of cortex, amygdala and hippocampus which causes the blood cortisol level to rise and the brain function to be affected through the neurons and the glucocorticoid receptors in the glial cells. This could be the possibility in the students who have taken up examination that the stress posed by them has activated the HPA axis and could have increased the secretion of salivary cortisol. Studies have reported an increase in salivary cortisol by 9 folds in students during examination than the students after examination [30]. Likewise, in another study the stress level of girls and boys, reported that significant increase of cortisol observed before the exams than after the exams [31]. Vivian NG *et al.*, has reported that a significantly high stress in students before exams than after the exams. It stated that the increase could be because of the students being aware of the deficiencies in subjects which may have stressed them. This anxiety and stress due to examination stress has shown a negative effect on their performance [32]. The various studies have indicated that wide range of prevalence of 7.7 - 65.5% for anxiety and stress among medical students substantially higher than general population. It was found that the prevalence of depression (45.3%) and anxiety (48.1%) were high during COVID -19 [33]. The anxiety during exams is likely to affect the performance and the anticipation to perform well could cause further stress to the students. These findings suggest that current medical education system may have deleterious effect on student's mental health with high frequency of depression, anxiety and stress.

## Conclusion:

The current study reported significant changes in moods parameters and cortisol level due to an examination stress in first year medical students. Since examination is an important criteria for students to prove themselves among all others, which may cause psychological distress in medical education. So, early identification and focus on student’s mental health is an obligatory need in the current CBME medical curriculum to achieve the competencies. Hence stress reduction interventions, orientation program in each year, evaluation process, how to cope, and how to get through each year without mental anguish are recommended. Starting a student counselling centre in the college with a qualified psychologist and peer advisor is also highly recommended. Student wellbeing not only develops a positive attitude to achieve their goals but also helps to achieve positive changes in the transition to adulthood.

## Funding:

None

## Ethics Approval and Consent to Participate:

All the procedures performed in these studies involving human participants were in accordance with the ethical standards of the institutional research committee and with the 1964 Helsinki declaration and its later amendments or comparable ethical standards. Consent was obtained from all participants included in the study.

## Figures and Tables

**Table 1:** Demographic and personal Characteristics of Study participants (n=150).

**Demographic characters of participants**
**Variables**		**Percent, %**
**Age**
17	26	17%
18	73	49%
19	33	22%
20	18	12%
**Gender**
Female	83	55%
Male	67	45%
**Year of study**
First year	150	100%
**Residents**
Hostel	96	64%
Day scholars	54	36%

**Table 2:** Classification of Depression, anxiety and Stress among the Study Population on the day of exam (Stressed) and before exam (relaxed state) assessed by DASS 10 scale (n=150).

**Variables**	**Relaxed (before exam)%**		**Stressed (day of exam)%**		**Male % (n=67)**		**Female% (n=83)**	
**Stress grading scale**
Low	108	72%	24	16%	15	10%	18	12%
Moderate	32	21%	84	56%	42	28%	36	24%
High	10	7%	42	28%	10	7%	29	19%
**Anxiety grading scale**
Low	125	83%	18	12%	25	37%	15	18%
Moderate	18	12%	96	64%	33	49%	38	46%
High	7	5%	36	24%	9	13%	30	36%
**Depression grading scale**
Low	138	92%	126	84%	54	81%	45	54%
Moderate	10	7%	18	12%	10	15%	26	31%
High	2	1%	6	4%	3	4%	12	14%
Total	150	100%	150	100%	67		83	

**Table 3:** Logistic Regression analysis of moods parameters, blood pressure and cortisol levels among study participants on the day of exam (Stressed) and before exam (relaxed) (n=150).

**Variables**	**Relaxed(beforeexam)**	**Stressed(dayofexam)**	**OR(95%CI)**	*p-value
	**Mean±SD**	**Mean±SD**		
Heartrate(beats/min)	72.52±1.09	77.32±2.52	1.923	0.091
SystolicBP(mmHg)	117±2.48	119±2.59	1.253	0.183
DiastolicBP(mmHg)	75.44±3.28	78.96±2.30	0.947	0.632
Stress	12.7±0.88	21.27±2.51	2.153	0.016*
Anxiety	11.27±1.40	24.06±4.01	3.038	0.0001**
Depression	6.88±0.6	10.23±1.09	2.513	0.041*
Cortisol(ng/ml)	2.61±0.40	5.14±0.35	2.872	0.0001**
Data presented are mean±SD and OR: Odds Ratio; 95% CI: 95% Confidence Interval. *Significant level indicates (*P<0.01, **P<0.001)

**Table 4:** Comparison of different correlates of stress, Anxiety, Depression and Cortisol in male and female students on the day of exam (Stressed) and before exam (relaxed) (n=150).

**Variables**	**Relaxed(beforeexam)**		**Stressed(dayofexam)**		*p-value
	**Male(67)**	**Female(83)**	**Male(67)**	**Female(83)**	
Stress	10.12±5.8	13.46±4.8	14.91±5.1	19.72±4.8	0.002**
Anxiety	7.42±4.5	11.01±3.5	16.82±3.8	21.27±5.1	0.001**
Depression	6.81±3.9	7.69±3.2	7.61±3.4	8.35±4.1	0.021*
Cortisol(ng/ml)	2.62±1.2	2.96±1.8	5.12±2.8	6.01±3.1	0.001**
Data presented are means±SD of stress, anxiety, depression and cortisol level among male and female in stressed and relaxed state. The statistical significant level indicates *P<0.01, **P<0.001.
